# Supplementary material for: Human antibody heavy chain variable region exon interacts with 3' regulatory region to form a stable somatic hypermutation center
Source: bioRxiv. 2026 Jul 22:2026.07.20.739632. Preprint. [Version 1] doi: 10.64898/2026.07.20.739632 (PMC13419765; doi:10.64898/2026.07.20.739632)
Supplement: Supplementary file 1 [file media-1.pdf]

Supporting Information for
Human antibody heavy chain variable region exon interacts with 3' regulatory region to form a stable somatic hypermutation center

Ying Guan[a,b,c,1], Mariia Mikhova[a,b,c], Zhuo Yang[a,b,c], Kai Xu[a,b,c], Shuxian Xie[a,b,c,2], Xin Lin[a,b,c], Adam Yongxin Ye[a,b,c], Taimor Williams[a,b,c], Jianshu Wang[d], David G. Schatz[d], Frederick W. Alt[a,b,c,*,†], Xi Chen[a,b,c,1,*]

[a]Howard Hughes Medical Institute, Boston Children's Hospital, Boston, MA 02115;

[b]Program in Cellular and Molecular Medicine, Boston Children's Hospital, Boston, MA 02115;

[c]Department of Genetics, Harvard Medical School, Boston, MA 02115;

[d] Department of Immunobiology, Yale School of Medicine, New Haven, CT 06520-8011;

1 Y.G. and X.C. contributed equally to this work.

2 Present address: University of California San Diego, La Jolla, CA 92093.

*Frederick W. Alt and Xi Chen.

†This study is conducted in Frederick W. Alt lab.

Email: frederick.alt@enders.tch.harvard.edu; Xi.Chen2@childrens.harvard.edu

This PDF file includes:

Figures S1 to S8
Tables S1 to S3

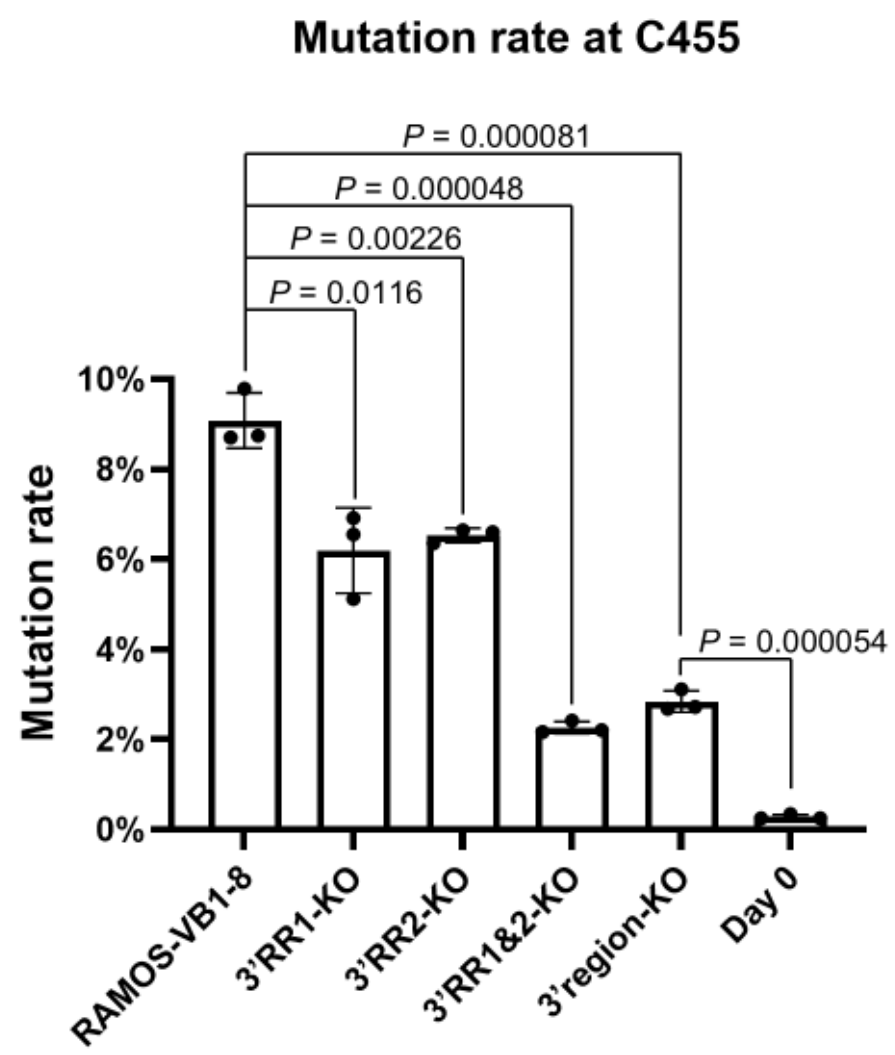

Fig. S1. Mutation frequency at the C455 nucleotide position of the VB1-8 exon in RAMOS-VB1-8, 3′RR1-KO, 3′RR2-KO, 3′RR1&2-KO, and 3′region-KO cells after 10 days of doxycycline treatment, as determined by SHM-Rep-Seq. The day 0 sample from untreated RAMOS-VB1-8 cells served as a negative control. Data are shown as mean ± SD from three independent repeats.

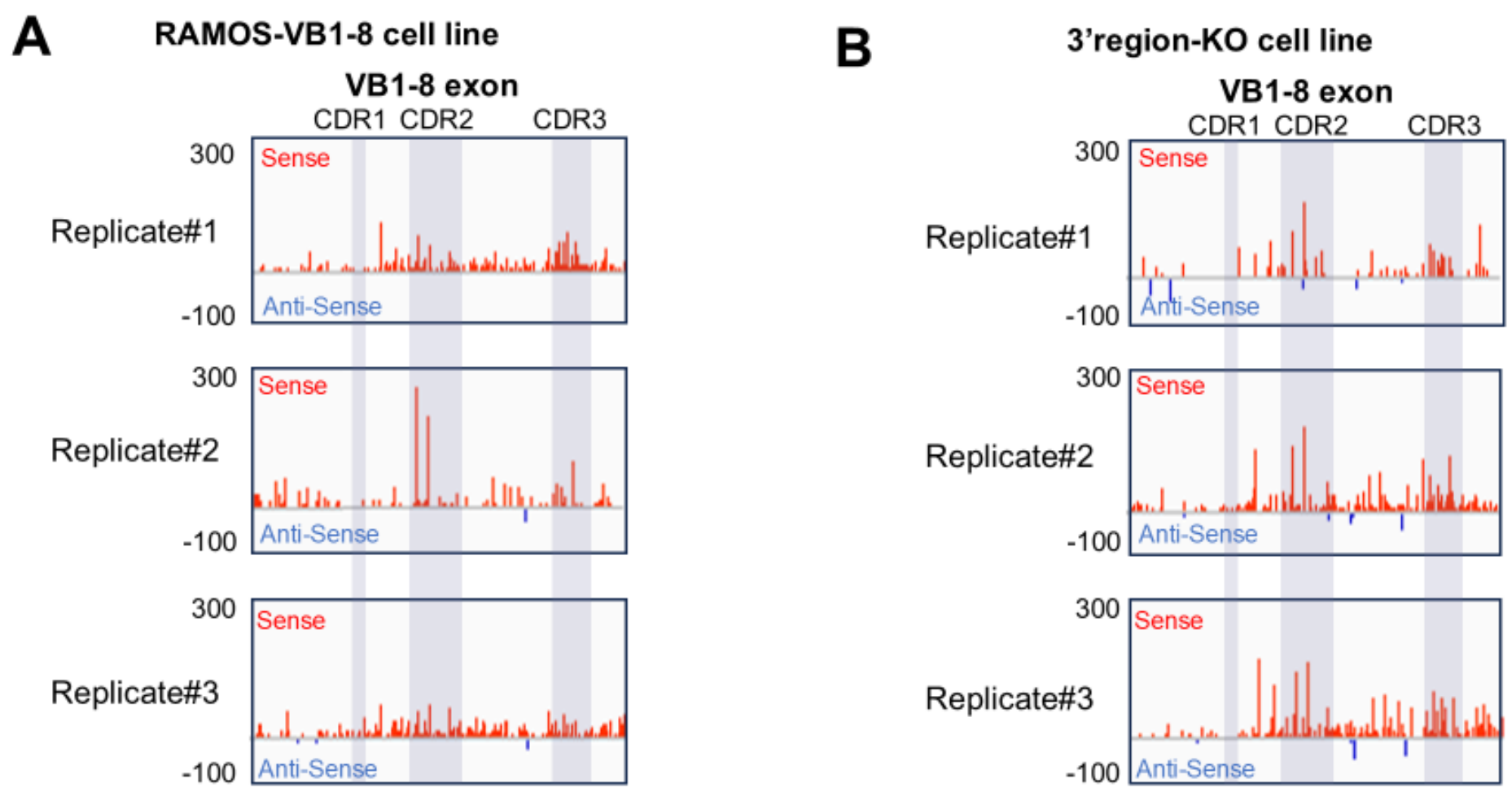

Fig. S2. Transcription levels of the VB1-8 exon in RAMOS-VB1-8 and 3′region-KO cells across independent replicates. (A,B) PRO-seq reads across the VB1-8 exon in RAMOS-VB1-8 cells (A) and 3′region-KO cells (B).

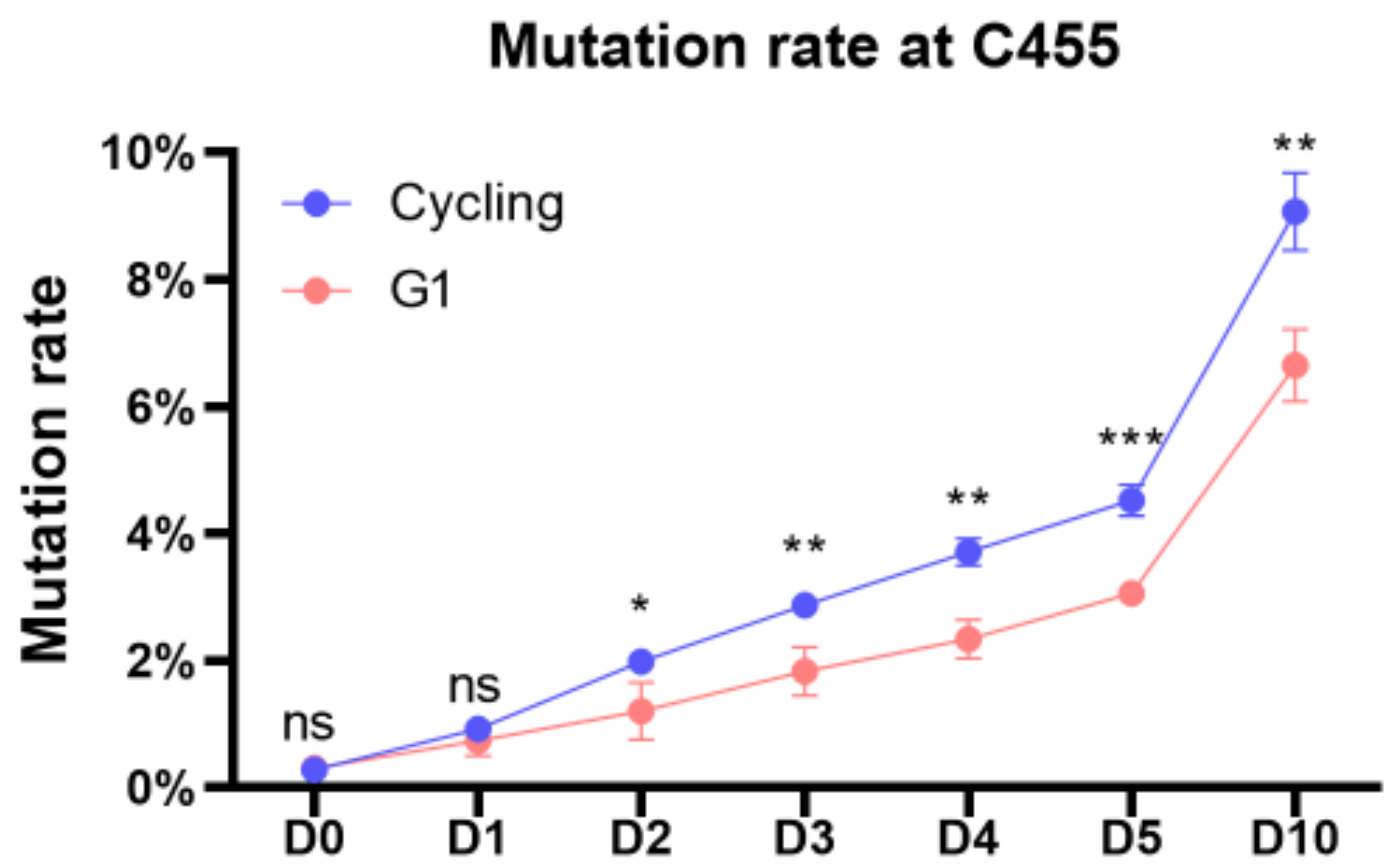

Fig. S3. Mutation rate at the C455 nucleotide position of the VB1-8 exon in cycling RAMOS-VB1-8 cells and G1-arrested RAMOS-VB1-8-Bcl2 cells, as determined by SHM-Rep-Seq. Data represent the mean ± SD of three independent repeats. * $P < 0.05$, ** $P < 0.01$, *** $P < 0.002$; ns, not significant.

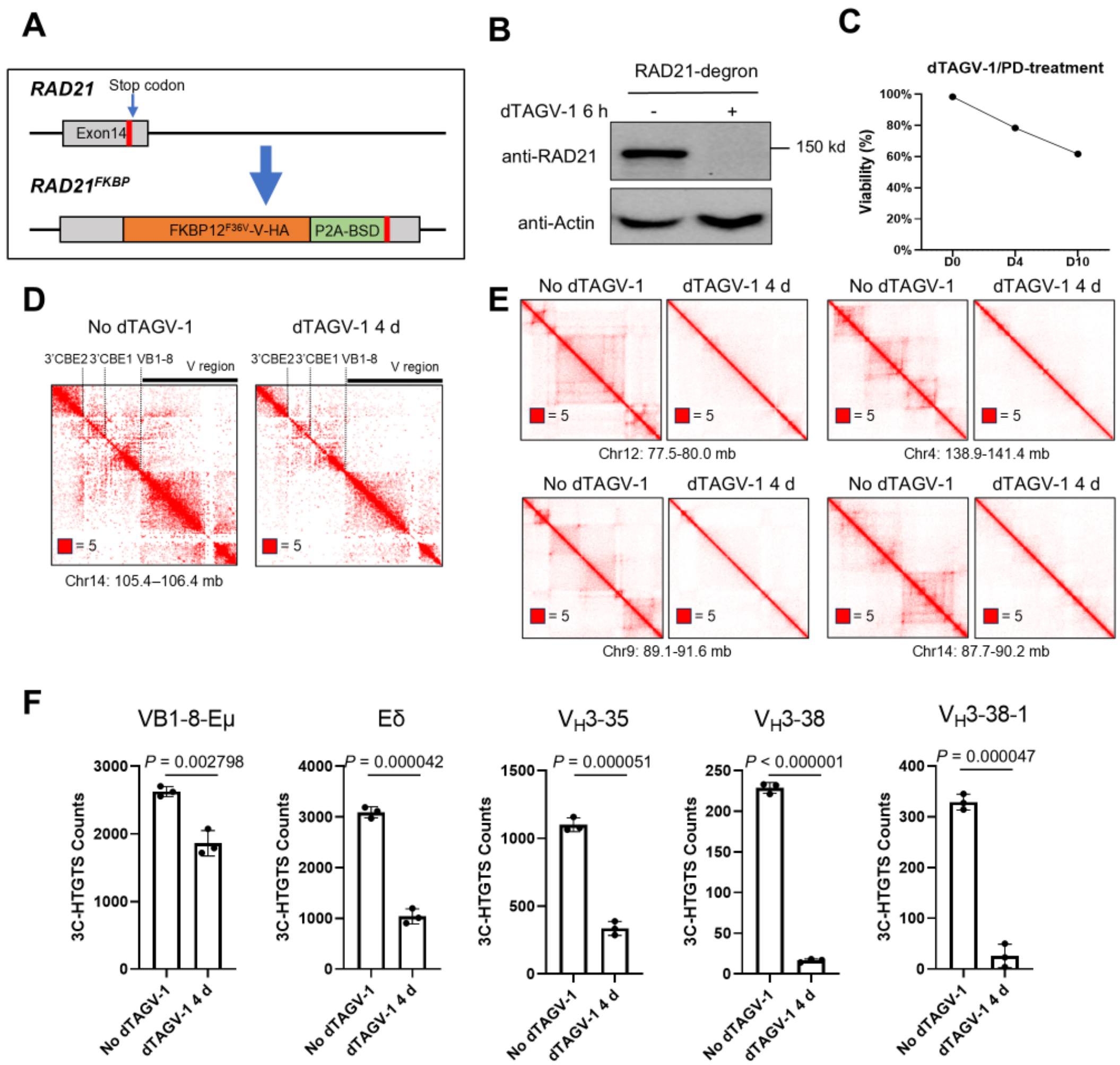

Figure S4. Validation of RAD21 depletion and its effects on chromatin architecture and transcription at the *IGH* locus. (A) Schematic of the strategy used to generate RAD21-degron cells. (B) Western blot analysis of RAD21 protein levels in RAD21-degron cells treated with or without dTAGV-1 for 6 hours. Raw data is shown in Figure S8B. (C) Cell viability of RAD21-degron cells following 10 days of dTAGV-1 treatment. Data represent the mean ± SD of three independent repeats. (D) Hi-C interaction maps of the *IGH* locus in RAD21-degron cells treated with or without dTAGV-1 for 4 days. (E) Hi-C interaction maps of four non-*IGH* loci in RAD21-degron cells treated with or without dTAGV-1 for 4 days. (F) Normalized 3C-HTGTS contact counts for each indicated 5-kb region from 3′RR-baited 3C-HTGTS libraries in RAD21-degron cells treated with or without dTAGV-1. Data are shown as mean ± SD from three independent repeats.

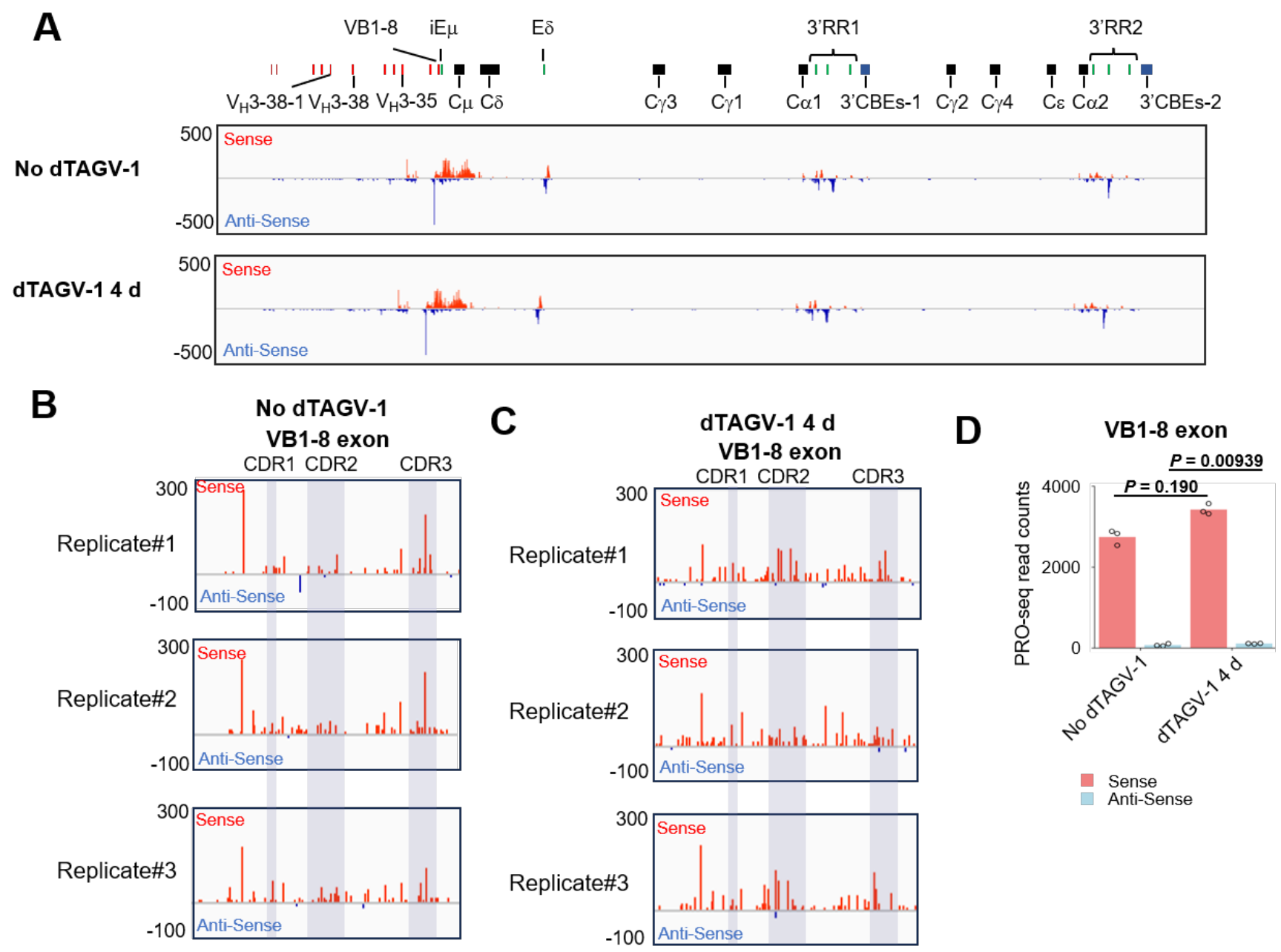

Figure S5. Transcription levels of the VB1-8 exon in RAD21-degron cells with and without dTAGV-1 treatment of independent replicates. (A-C) Normalized PRO-seq read counts across the *IGH* constant region (A) and the VB1-8 exon (B-C) in RAD21-degron cells treated with or without dTAGV-1 for 4 days. (D) Quantification of normalized PRO-seq read counts across the VB1-8 exon in RAD21-degron cells treated with or without dTAGV-1 for 4 days. Statistical significance was assessed using a two-tailed unpaired Student's t-test. Data represent the mean of three independent repeats.

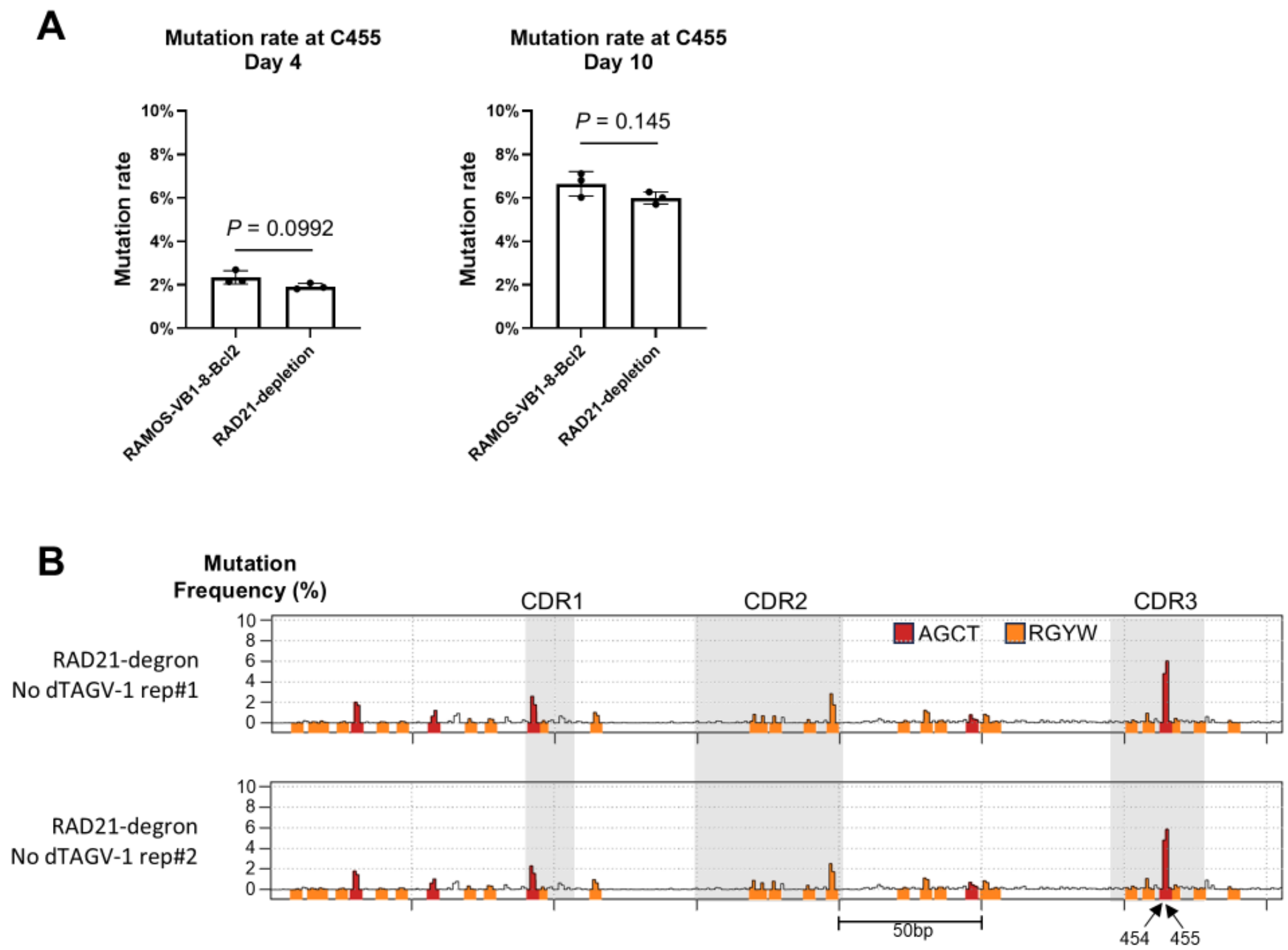

Figure S6. VB1-8 mutation efficiency in RAD21-degron cells. (A) Mutation frequency at the C455 site in RAMOS-VB1-8-Bcl2 and RAD21-depleted cells after 4 or 10 days of doxycycline treatment, as determined by SHM-Rep-Seq. Data represent the mean ± SD from three independent repeats. (B) SHM profiles across the VB1-8 exon in RAD21-degron cells following 10 days of doxycycline-induced AID expression without dTAGV-1 treatment, as determined by SHM-Rep-Seq. Data are shown from two independent replicates.

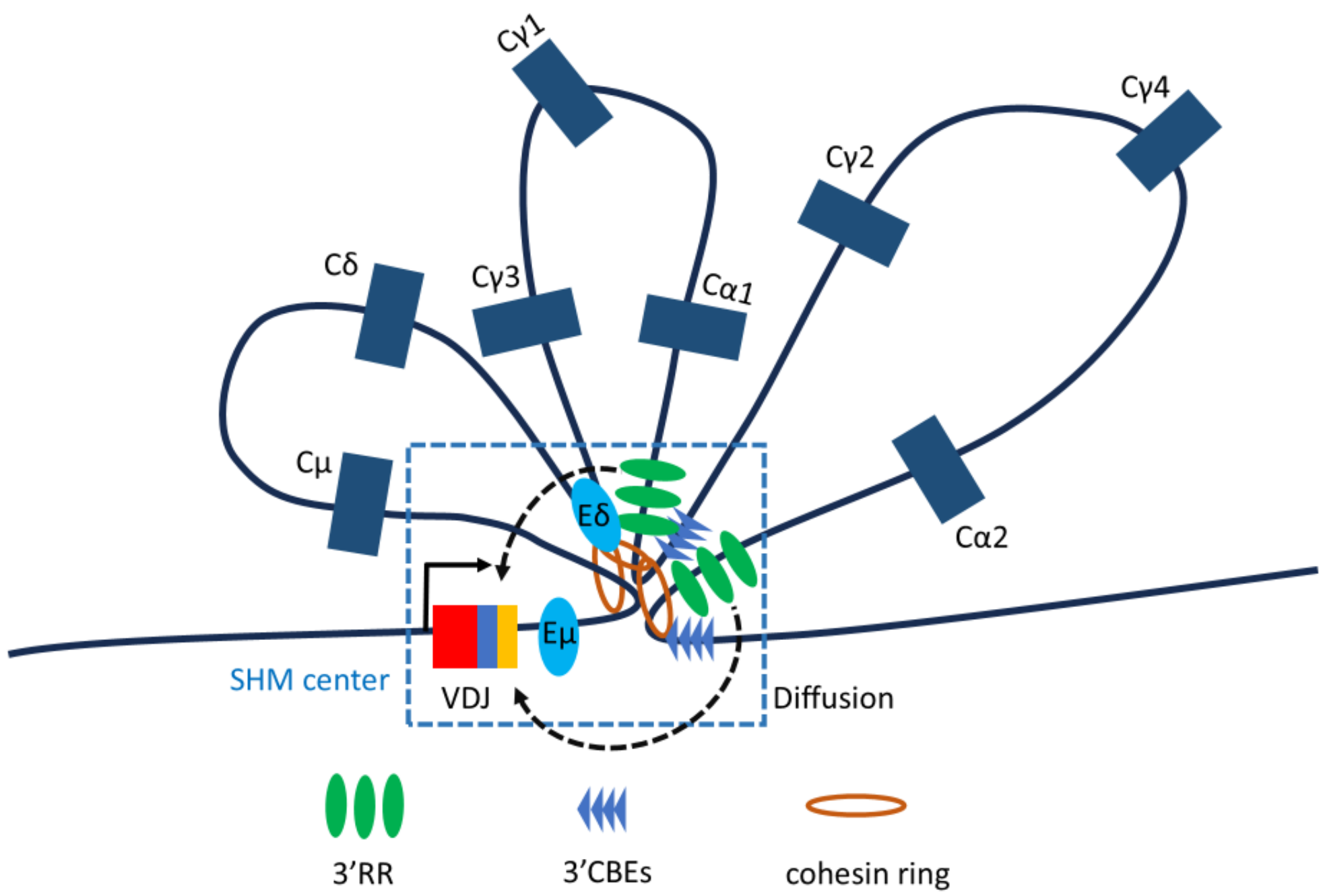

Figure S7. This figure depicts one model for the structure of the proposed SHM-C, based on the data described in the manuscript. We propose that in GC B cells that cohesin is loaded within each set of C_H exons sequences, potentially within the 3'RRs and/or the upstream Eμ/Sμ region. Extrusion through the upstream C_H exons would be unimpeded but then dynamically impeded by the transcribed iEμ/Sμ sequences upstream and the transcribed 3'RR1/3'CBE1 downstream to form a dynamic upstream loop that juxtaposes the VDJ and 3'RR1/3'CBE1 in one domain of the SHM center. Extrusion from cohesin loaded in the downstream C_H/3'RR sequences would be dynamically impeded the transcribed 3'RR2/3'CBE2 downstream and by the 3'RR1/3'CBE1 upstream to form a dynamic downstream loop that juxtaposes 3'RR2 and 3'RR1 to establish the second domain of the SHM center. This model would position both 3'RRs within short-range diffusion distance from the V(D)J exon and thereby able to support SHM activity. Both the upstream-based and downstream-based downstream domains could form an active 3'RR1-based or 3'RR2-based SHM-C in the absence of the other in the context of this model, explaining our finding that one or the other 3'RR can support SHM. Please see text or the Supplementary Discussion of reference 13 for more details.

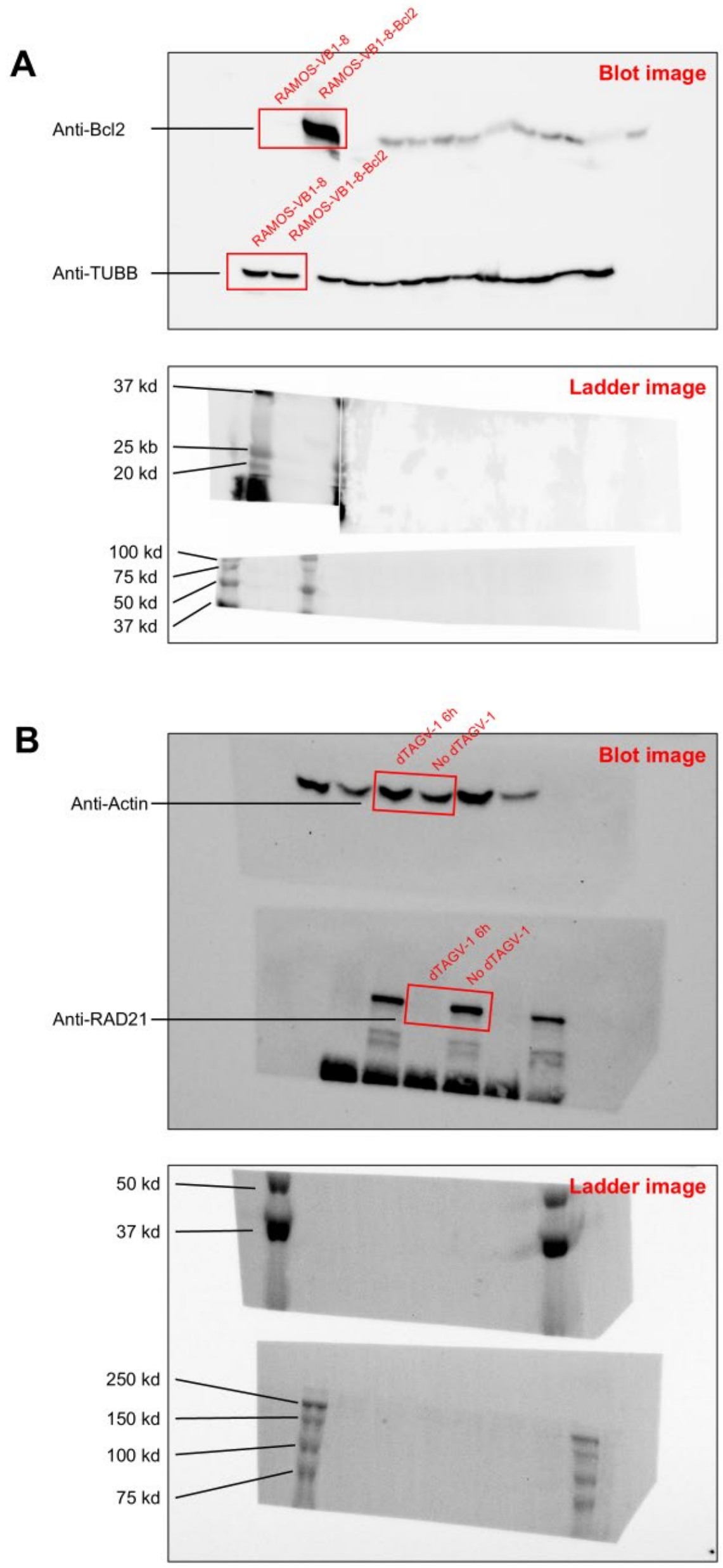

Figure S8. Unprocessed western blot analysis. (A) Bcl2 western blot for RAMOS-VB1-8 and RAMOS-VB1-8-Bcl2 cells. Top, blot image; bottom, ladder image. Red boxes indicate the regions presented in Figure 4D. The blot was generated from the same membrane, which was cut prior to probing with different antibodies. (B) RAD21 western blot for RAD21-degron cells treated with or without dTAGV-1 for 6 hours. Top, blot image; bottom, ladder image. Red boxes indicate the regions presented in Figure S4B. The blot was generated from the same membrane, which was cut prior to probing with different antibodies.

Tables

Table S1. List of sgRNAs

Name	Sequence	Purpose
sgRNA-Ramos VDJ 5'-2	CGGCGTGTAGACATCTACGT	Targeting the endogenous VDJ locus upstream, for VB1-8 insertion
3gRNA-vb18p-new1	CAATGCTCAGGAAACCCCAC	Targeting the endogenous VDJ locus downstream, for VB1-8 insertion
RRCBE-sg4	CCGACCCGCCCTTACTCACG	Targeting the upstream of 3'RR-2
RRCBE-sg5	GTGGAGATATGCGGAAGTGT	Targeting the downstream of 3'RR-2
RRCBE-sg7	GCCTTCCAGGTGCAATAAAG	Targeting the upstream of 3'RR-1
RRCBE-sg2	GTGAAGGTGTGAGAGTGTGA	Targeting the downstream of 3'RR-1
grna-rr1cbe1	CTGCAGGTCACTTACTCATG	Targeting the upstream of 3' region
grna-rr2cbe2	GACGTCCTGACCACCCTAAT	Targeting the downstream of 3' region
AAVS1 sgRNA-4	GGGGCCACTAGGGACAGGAT	Targeting the AAVS1 locus, for Bcl2 expression cassette insertion
sgRAD21	CCAAGGTTCCATATTATATA	Targeting the RAD21 locus, for FKBP tag insertion

Table S2. List of oligos.

Name	Sequence	Purpose
bio-vb18-F3	ACATATATATGGGTGACAATGACATC	bio primer for VB1-8 bait for SHM-Rep-seq and 3C-HTGTS
red-vb18	AATGACATCCACTTTGCCTTTCTCT	red primer for VB1-8 bait for SHM-Rep-seq
VB18-red-3C	GGCCACACTGACTGTAGACAAAC	red primer for VB1-8 bait for 3C-HTGTS
bio-HS12-loca1-F1	GTCCTGGTCCCAAAGATGGC	bio primer for 3'RR bait for 3C-HTGTS
red-HS12-loca1	AAGCTATTTCTGGAAACAGCCTCAG	red primer for 3'RR bait for 3C-HTGTS

Table S3. List of antibodies.

Name	Source	Purpose and dilution ratio
Anti-Bcl2	Abcam, # ab182858	Western blot, 1:2000
Anti-TUBB	Abcam, # ab78078	Western blot, 1:1000
Anti-RAD21	Abcam, # ab154769	CUT&RUN, 1:100
Anti-RAD21	Abcam, # ab992	Western blot, 1:1000
Anti-Actin	CST, # 5125	Western blot, 1:4000
Anti-AID	Home made	CUT&RUN, 1:100
